# Assessment of an Integrated and Sustainable Multistage System for the Treatment of Poultry Slaughterhouse Wastewater

**DOI:** 10.3390/membranes11080582

**Published:** 2021-07-30

**Authors:** Phumeza Akhona Dyosile, Cebisa Mdladla, Mahomet Njoya, Moses Basitere, Seteno Karabo Obed Ntwampe, Ephraim Kaskote

**Affiliations:** 1 Bioresource Engineering Research Group (BioERG), Cape Peninsula University of Technology, Symphony Way, Bellville Campus, Cape Town 7535, South Africa; phumezadyosile@gmail.com (P.A.D.); mdladlacebisa44@gmail.com (C.M.); mahomet.njoya@gmail.com (M.N.); 2 Academic Support Programme for Engineering in Cape Town (ASPECT) & Water Research Group, Department of Civil Engineering, University of Cape Town, Rondebosch, Cape Town 7700, South Africa; 3 Centre of Excellence in Carbon-Based Fuels, School of Chemical and Minerals Engineering, North West University, Private Bag X1290, Potchefstroom 2520, South Africa; Karabo.Ntwampe@nwu.ac.za; 4 Malutsa (Pty) Ltd., c/o Oude Pont and Meent Street (Malutsa House), Wellington Industrial Park, Wellington 7655, South Africa; ephraimk@malutsa.co.za

**Keywords:** down-flow expanded granular bed reactor (DEGBR), expanded granular sludge bed (EGSB), membrane bioreactor (MBR), poultry slaughterhouse wastewater (PSW)

## Abstract

This paper assesses the performance of an integrated multistage laboratory-scale plant, for the treatment of poultry slaughterhouse wastewater (PSW). The system was comprised of an eco-flush dosed bio-physico pre-treatment unit for fats, oil, and grease (FOG) hydrolysis prior to the PSW being fed to a down-flow expanded granular bed reactor (DEGBR), coupled to a membrane bioreactor (DEGBR-MBR). The system’s configuration strategy was developed to achieve optimal PSW treatment by introducing the enzymatic pre-treatment unit for the lipid-rich influent (PSW) in order to treat FOG including odour causing constituents such as H_2_S known to sour anaerobic digestion (AD) such that the PSW pollutant load is alleviated prior to AD treatment. This was conducted to aid the reduction in clogging and sludge washout in the DEGBR-MBR systems and to achieve the optimum reactor and membrane system performance. A performance for the treatment of PSW after lipid reduction was conducted through a qualitative analysis by assessing the pre- and post-pre-treatment units’ chemical oxygen demand (COD), total suspended solids (TSS), and FOG concentrations across all other units and, in particular, the membrane units. Furthermore, a similar set-up and operating conditions in a comparative study was also performed. The pre-treatment unit’s biodelipidation abilities were characterised by a mean FOG removal of 80% and the TSS and COD removal reached 38 and 56%, respectively. The final acquired removal results on the DEGBR, at an OLR of ~18–45 g COD/L.d, was 87, 93, and 90% for COD, TSS, and FOG, respectively. The total removal efficiency across the pre-treatment-DEGBR-MBR units was 99% for COD, TSS, and FOG. Even at a high OLR, the pre-treatment-DEGBR-MBR train seemed a robust treatment strategy and achieved the effluent quality set requirements for effluent discharge in most countries.

## 1. Introduction

Millions of litres of poultry slaughterhouse wastewater (PSW) are generated annually from a series of process steps used for bird processing for meat, which includes the slaughtering process, meat handling, and cleaning of facilities and equipment [1]. From this, the contaminated wastewater contains a high concentration of organic matter from blood, skin, fats, including nutrients and the chemical pollutants from detergents used to clean holding facilities, bird urine, and faecal matter, thus, making the disposal of this high-strength wastewater perilous [1,2]. Beyond the risks linked to the deterioration of the environment, pollution is one of the most pressing environmental health priorities requiring intervention. Similarly, the strength of PSW has been proven to be much higher than domestic wastewater and reduces water-dissolved oxygen, thus, affecting water-dependent species when released into receiving sources untreated [3,4]. While poultry slaughterhouses have employed wastewater treatment units, most are outdated and incapable of achieving suitable contaminant and nutrient reduction as per the set regulations. Overtime, the discharged wastewater may result in sewer line damage, the contamination of water bodies, the pollution of land, and the possible emission of harmful gases from the nutrients and contaminants contained therein [5].

Furthermore, the urgency for effective treatment methods for PSW lie not only in the need for continued wastewater treatment research but can have financial benefits due to the escalating growth demand for poultry products that is aided by the South African agro-economy expansion. However, for such growth to be sustainable, the devastating effect the wastewater has on the environment, growing water use, and demand, water conservation and recycling should be implemented worldwide. Hence, in the efforts to conserve water reserves in an already stressed water scarce country, i.e., South Africa (SA), researchers have since employed various technologies in the pursuit of effective PSW treatment options.

All around the world, biological treatment systems have been explored for PSW treatment due to their efficiency, i.e., nutrient and organic matter removal abilities, using several technologies. Furthermore, these systems are appreciated for their simplicity and reduced hydraulic retention times (HRTs) while handling high organic loading rates (OLRs) [6]. These biological systems contain anaerobic, aerobic, or facultative microorganisms that degrade organics in the PSW [7,8]. However, others [9] substantiated the superiority of anaerobic digestion in comparison to aerobic digestion as a secondary treatment unit, whereby an aerobic system was compared to an anaerobic system in a comparative study using a series of case studies that looked at the up-flow anaerobic sludge blanket (UASB), an activated sludge process, among others. In addition, the study concluded that aerobic systems are recommended for post-treatment, particularly in bigger plants for nutrient removal and further PSW purification to meet the general limit for effluent discharge than as primary systems for PSW [9,10]. Furthermore, some [10] continued and attested aerobic technologies as better post-treatment technologies to best treat the solubilised organics in FOG laden effluent, utilising a system comprised of an anaerobic baffled reactor (ABR) seeded with activated sludge (AS). The study’s observations attested to additional organic reduction from 128 and 132 mg/L to 0.1 and 0.4 mg/L for total organic carbon (TOC) and COD, respectively. In another research study by [11], an anaerobic–aerobic system using an EGSB coupled with a membrane bioreactor (MBR) was evaluated. The bioreactor achieved a COD average removal of 63% and, when combined with a MBR, an average of 96% removal was achieved, thus signifying that the anaerobic–aerobic set-up can be effectively used for PSW treatment. This was substantiated by [10], using a concept of pre- and-post-treatment.

The anaerobic process has limitations such as high temperature sensitivity, inability to efficiently remove nutrients, and depending on the configuration and operating conditions, clogging of the granular bed due to solids and FOG settling over the bed. Additionally, low to moderate effluent quality and longer start up periods from the acclimation period for the organic matter decomposing microorganisms, were identified as the main drawbacks [12]. Table 1 lists a summary of technologies used and the achieved COD, TSS, and FOG removal efficiency using the anaerobic reactors for the lipid-rich PSW treatments to date.

Despite the positive advances taken, several researchers found that high protein wastewater (such as PSW) results in the production of free ammonia during anaerobic digestion [13]. They recommended a pre-treatment stage to treat the wastewater characteristics for enhanced anaerobic digestion (AD) performance without producing detrimental by-products such as ammonia. Additionally, the authors of [10] indicated that anaerobic processes require pre- and post-treatment steps for the appropriate removal of solids, nutrients, and FOG that tend to clog the AD reactor and piping system thereof, resulting in a reduced reactor performance and an accelerated reactor failure. Furthermore, in up-flow reactors, periodic sludge washout due to suspended solids and high lipid content has been observed [1,12].

To date there have been several pre-treatment methods developed from the findings conducted for AD. Table 2 lists a summary of the effective pre-treatment methods coupled with AD in numerous studies. From the results, it is also evident that a pre-treatment unit can remove some of the contaminants.

Similarly, there are post-treatment technologies that can be used, among which membrane bioreactors are the most researched. These reactors sometimes consist of activated sludge and membranes that contain distinct pores that remove dissolved organic and inorganic contaminants by providing a physical barrier that filters out pollutants and bacteria [19]. Many studies have included membrane bioreactors as a tertiary treatment stage achieving exceptional results as high as 98–>99% removal efficiency [19,20].

**Figure 1:**
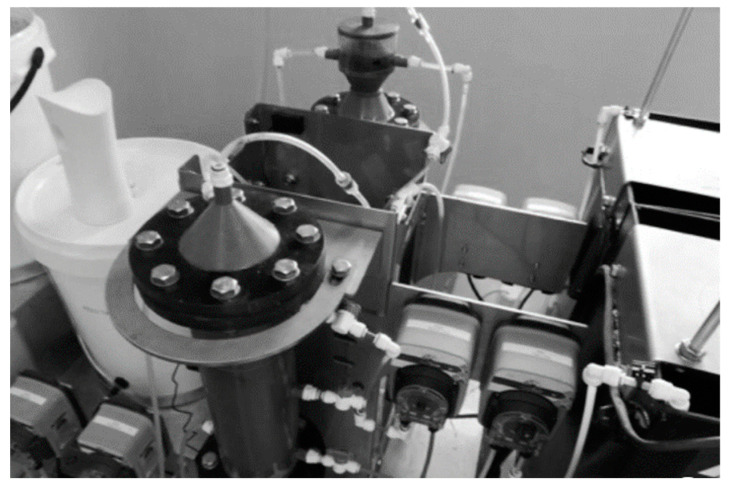
Lab-Scale Treatment Plant for PSW [21]—Reprinted from Membranes, vol 11, Meyo, H.B.; Njoya, M.; Basitere, M.; Ntwampe, S.K.O.; Kaskote, E., “Treatment of Poultry Slaughterhouse Wastewater (PSW) Using a Pre-treatment Stage, an Expanded Granular Sludge Bed Reactor (EGSB), and a Membrane Bioreactor (MBR)”. Copyright (2021), with permission from MDPI.

**Figure 2:**
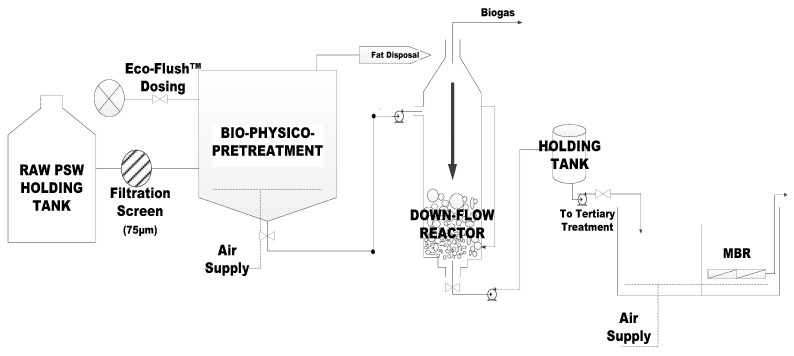
Process Flow Diagram of the Treatment Plant.

Similarly, post AD, a membrane unit was installed for the further purification of the formed by-products during the AD process [8], subsequently assisting in the further remediation of the wastewater. Submerged membranes are well known for fouling primarily in high FOG wastewater such as that from poultry slaughterhouses. As per the Membrane Bioreactor Task Force of the Water Environment Federation (WEF) (2012), MBR requires coarse influent screening, grit removal, fine screening, and primary clarification, all of which are pre-treatment technologies. Therefore, studying the effectiveness of a combined pre-treatment, an AD bioreactor, and a submerged membrane system is, therefore, crucial for this study. As shown in Figure 1 and Figure 2, a set-up as illustrated was used in this study for the treatment of PSW.

From previous studies, the recommendations included a thorough pre-treatment step to rid the PSW of solidified FOG in order to enhance the AD reactor’s performance [1,22]. From the identified gaps, this research included a performance review on a biological pre-treatment stage using a commercial product, i.e., Eco-flush^TM^ as an additive providing active enzymes for FOG hydrolysis. With the pre-treatment process in place, this study further investigated the performance of the treatment of PSW after the FOG reduction in the pre-treatment unit. Furthermore, the efficacy of the MBRs as a post treatment technology was also investigated.

Lastly, the performance of the full pre-treatment, AD, and MBR chain was investigated.

## 2. Materials and Methods

### 2.1. PSW Sampling

PSW was collected from a local abattoir in the Western Cape, SA. The sample was drawn from an in-between slaughtering process and wastewater processing stage to acquire a representative raw PSW sample. For preservation, the wastewater was stored in a temperature-controlled unit at 5 °C. A representative sample was taken to study the COD, TSS, and FOG of the raw incoming PSW in comparison to the established raw PSW average conditions, as shown in Table 3.

### 2.2. Experimental Set-Up

The set-up configuration is as shown in Figure 2. All the units had biogas collection ports, although this did not form part of this study.

#### 2.2.1. Pre-Treatment Unit Set-Up

The PSW was pre-treated by dosing 20 mL of Eco-Flush^TM^, supplied by Mavu Biotechnologies (Pty) Ltd., (Cape Town, South Africa) into a 20-litre pre-treatment tank with raw PSW. The Eco-flush^TM^ contained a complex mixture of microorganisms, including aerobic, anaerobic, nitrifying, and sulphur oxidising bacteria combined with fungi and enzymes associated with the hydrolysis of FOG, i.e., the dissociation of bonds between the triglycerides and phospholipase resulting in a glycerol and fatty acid separation. Furthermore, not only does Eco-flush^TM^ aid the reduction in FOG content in PSW, it also contains a bacterial mixture that synergistically produces enzymes (in a nutrient rich environment) that accelerate the decomposition of organic matter and oxidation of ammonia into nitrates and nitrites [25].

**Figure 3:**
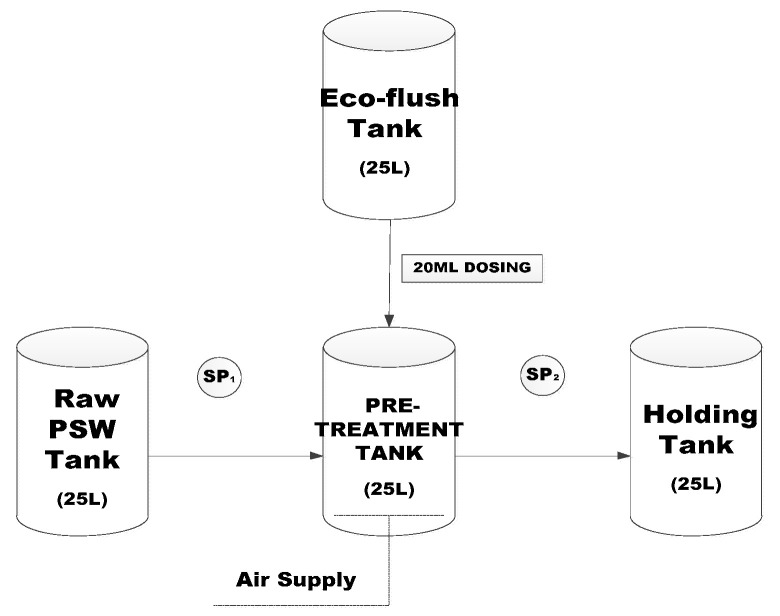
Pre-treatment Experimental Set-up (SP = sample point).

A Resun air pump (Ac 9906) from Hydroponic in Cape Town, South Africa, was used to sparge air into the pre-treatment tank using silicone tubing. The silicone tubing used to pump air into the pre-treatment tank was connected to two diffusers to provide sufficient micro-bubble formation into the system. This ensured that there was an adequate dissolved air supply to create the optimum conditions for aerobic bacteria to be most effective. The mixture was aerated for 24 h then allowed to settle for a further 24 h to allow the Eco-Flush^TM^ time to adequately digest the FOG and decouple proteins within the PSW and to reduce the level of H_2_S, which is known to deactivate anaerobic bacteria. The pre-treated PSW was then filtered with a 75-micrometre Madison Test sieve into a pre-treatment tank, which feeds into both the EGSB (previous study and for comparison) and DEGBR. The PSW to the AD systems was continuously stirred with a stirrer to keep the feed in a homogeneous state. The treatment process is depicted in Figure 3.

#### 2.2.2. Bioreactor Experimental Set-Up

**Figure 4:**
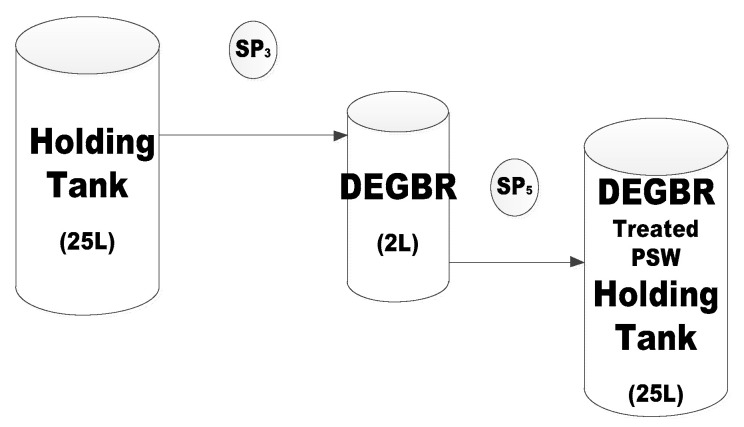
Reactor (EGSB and DEGBR) Set-up. (SP = sample point).

Laboratory scale (2 L) Polyvinylchloride (±600 × 110 cm) DEGBR and EGBS reactors, were simultaneously set up parallel to each other, as illustrated in Figure 4. Each reactor was connected to a feed holding tank containing pre-treated filtered feed from the Eco-flush^TM^ pre-treatment unit (Illustrated in Figure 4).

The reactors were inoculated with anaerobic granules from an USAB operated at a local brewing house situated in Newlands, Cape Town. A volume (200 mL) of 50% v/w dry milk was used as a source of carbon and was fed to the granules, to assist with the acclimation of the anaerobic biomass.

For optimal acclimation, mesophilic temperatures (29–36 °C) were maintained by connecting an external water bath to the reactors jacket. Reactor inoculation took a period of 72 h prior to running the reactors at 0.36 L/h and had an HRT of 5.71hr. Product released was stored in separate holding tanks for individual performance analysis conducted at an SANAS-accredited external laboratory, i.e., City of Cape Town Scientific Services.

#### 2.2.3. Membrane Bioreactor Units Set-Up

- Membrane Bioreactor design

**Figure 5:**
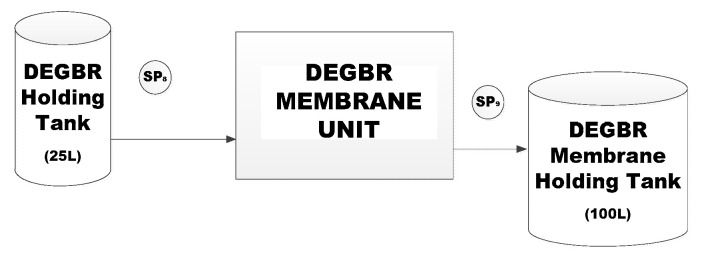
Membrane Bioreactor Unit Set-up. (SP=sample point).

Hydrophilic polyethersulfone (PES) membranes with a 0.04-mirometre pore size and a glycerine (20%)/sodium benzoate (3%) preservative were housed in a 315 × 182 × 69 mm polyvinyl chloride (PVC) module with a polyester (PET) drainage layer. The casts were submerged in 100 L aerated tanks, as illustrated in Figure 5.

- Inoculation and Operating Conditions

The EGSB-MBR and DEGBR-MBR had identical set-up conditions. A volume (25 L) of AD-treated PSW was filled in each tank. The aerated MBR systems acclimatised for a period of 48 h prior to the filtration cycle start-up at low flow rates of 0.36 L/h. The observed operated conditions included a temperature range of 5–40 °C and pH range of 2–11.

### 2.3. Sampling Points and Analysis

The sample point is as demonstrated in Figure 3 as SP_2_. Samples of the pre-treated PSW were taken every second day, while sampling points for the DEGBR were collected from SP_3_ and SP_5_, respectively, as illustrated in Figure 4. After the MBR was inoculated, the samples for the DEGBR-MBR systems were collected weekly at SP_9_, as shown in membrane set-up unit (Figure 5).

Once collected, the samples were stored at cooling storage at 5 °C to avoid acidification prior to analysis. See Table 4 for methods.

## 3. Results and Discussion

### 3.1. Pre-Treatment Performance Evaluation

**Figure 6:**
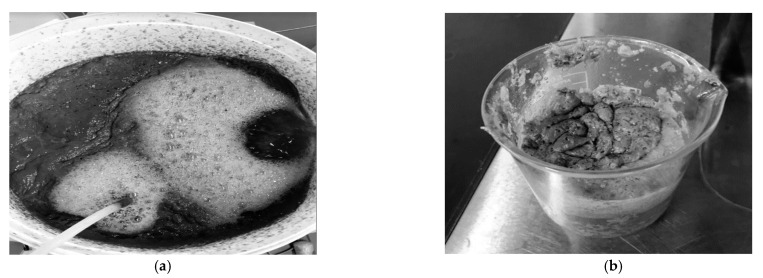
(**a**) During pre-treatment; (**b**) Collected coagulated FOG after pre-treatment. (**a**) Eco-flush^TM^ dosed pre-treatment during and after 24 h aeration when the enzymatic pre-treatment is employed, (**b**) The coagulated FOG collected from the top of the pre-treatment unit.

The development of the treatment plant used for this study included the Eco-flush^TM^ dosed bio-physico aerated pre-treatment unit that encouraged FOG coagulation and hydrolysis, which reduced the risk of FOG accumulation in the reactor bed and piping system [25]. After the 24-h aeration period, the enzyme activity had facilitated the effective separation of glycerol from the lipid-rich PSW through hydrolysis, thus, corroborating research from other studies [13], whereby it was concluded that hydrolysis promotion was evident in the Eco-flush^TM^ dosed pre-treatment unit, hypothesised to be facilitated through lipase action. Figure 6 depicts the pre-treatment operation and FOG collected after coagulation.

**Figure 7:**
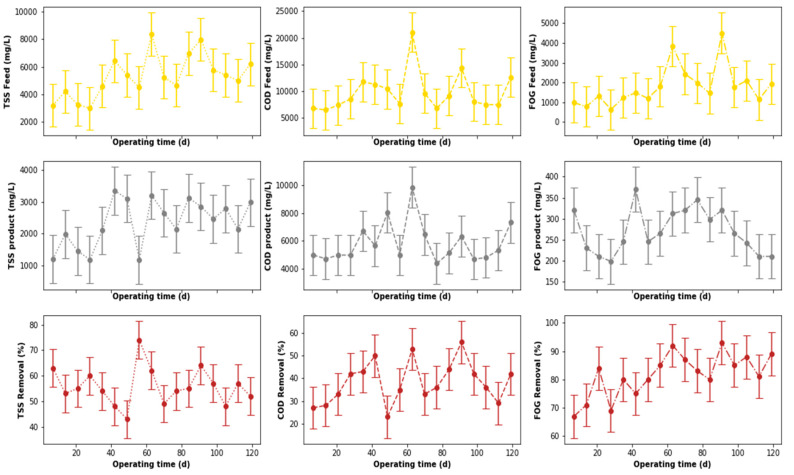
Performance of PSW pre-treatment stage before outliers’ detection and replacement.

**Figure 8:**
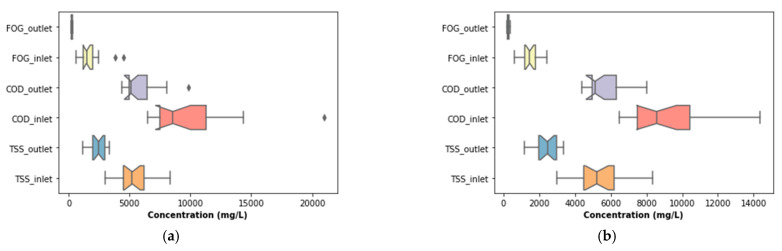
Boxplots of highlighted features before and after outliers’ replacement: (**a**) Boxplots before outliers’ replacement; (**b**) Boxplots after outliers’ replacement.

The performance of the pre-treatment stage used in this study is illustrated in Figure 7, which depicts the variation of key water quality assessment parameters as well as their removal efficiencies throughout the study. These parameters were not pre-processed to exclude outliers that may have been identified during the experiment. To identify these outliers, boxplots (see Figure 8a) were used. According to Figure 8a, outliers were identified in the distribution of the inlet FOG and COD, as well as the outlet COD. These outliers can be less clearly observed in Figure 7, which depicts the variation of these parameters throughout the study. To identify these outliers, the interquartile range rule was used, and they were, subsequently, replaced by the median value of respective distribution to correct the effects of the outliers.

**Figure 9:**
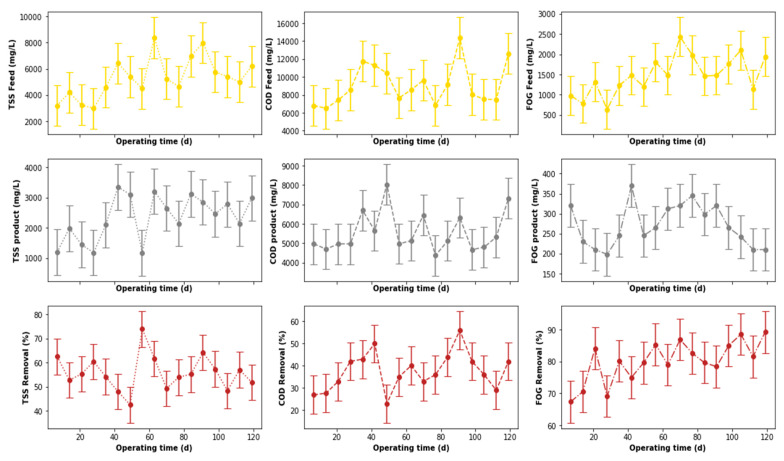
Performance of PSW pre-treatment stage after outliers’ detection and replacement.

**Figure 10:**
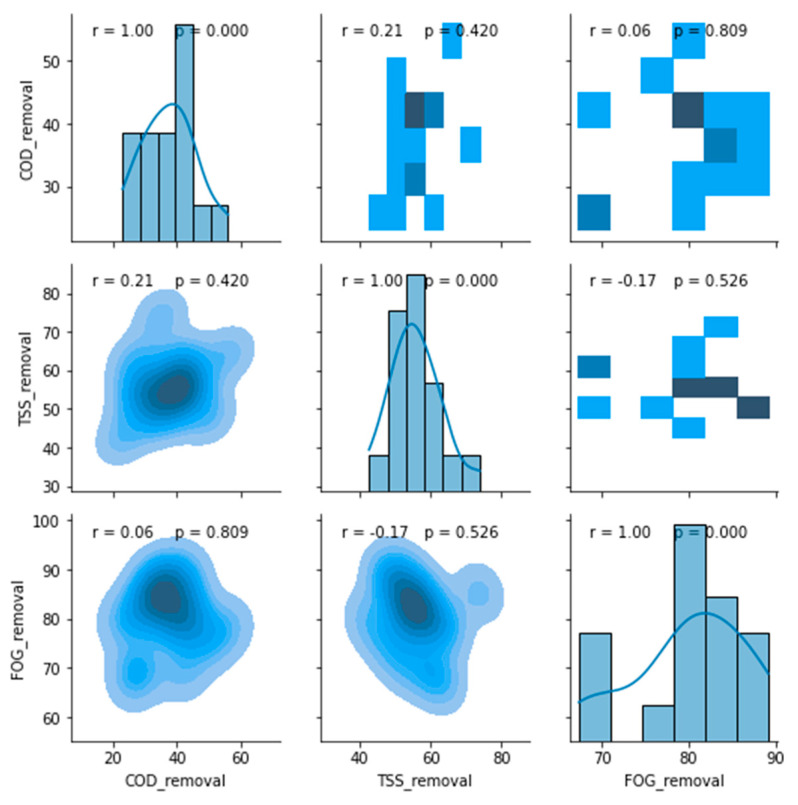
Correlation matrix between removal efficiencies of the pre-treatment stage.

The boxplot redacted performance profile of the PSW pre-treatment stage is displayed in Figure 9, from which it can be observed that this treatment stage performed well for the removal of FOG (65 to ~92%) and TSS (~45 to ~72%), with an insignificant removal of COD (~25 to ~52%) in comparison to the literature [13,16,17]. An evaluation of a possible correlation between these removal efficiencies was conducted in Figure 10, which depicts a correlation matrix with Pearson’s correlation coefficient (r) and the *p*-value for hypothesis testing. Usually, an R-value above 0.75 translates to a considerable correlation between the assessed parameters, which can be confirmed with a *p*-value ≤ 0.05. However, the removal values displayed in Figure 10 showed no correlation between the COD, FOG, and TSS removal efficiencies for the pre-treatment unit.

**Figure 11:**
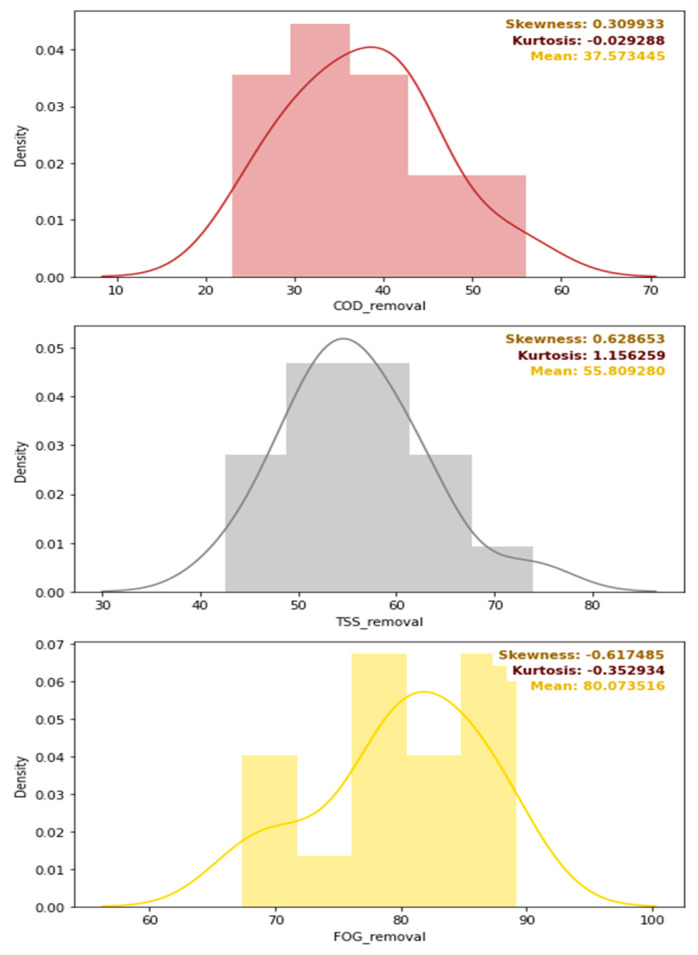
Density distribution, skewness, kurtosis, and mean values of the removal efficiencies of the pre-treatment stage.

A further evaluation of the distribution of the removal efficiencies is depicted in Figure 11, which provides the density distribution, the skewness, the kurtosis, and the mean of the COD, TSS, and FOG distributions. Overall, as initially intended, the pre-treatment stage performed the best for FOG removal as designed, with a mean FOG percentage removal of 80% (see Figure 11), while the mean percentage removal of the COD and TSS, were 38 and 56%, respectively. The skewness of each of these distributions was low, but varied with different kurtosis values, as depicted in Figure 11.

### 3.2. DEGBR Treatment Performance

**Figure 12:**
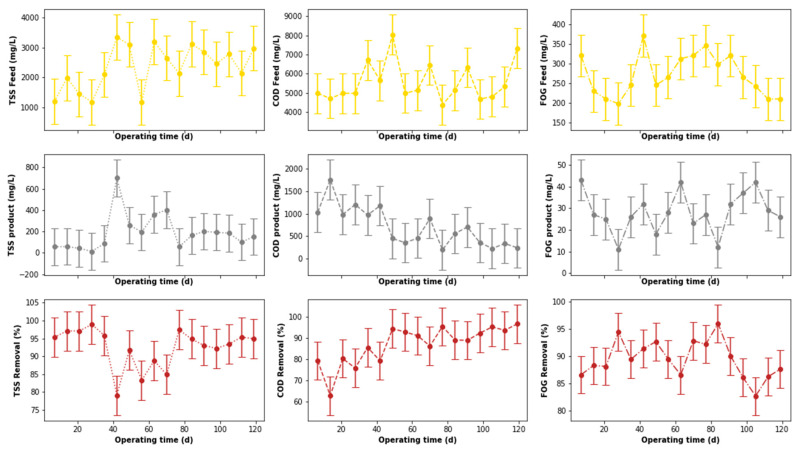
Performance of PSW DEGBR before outliers’ detection and replacement.

**Figure 13:**
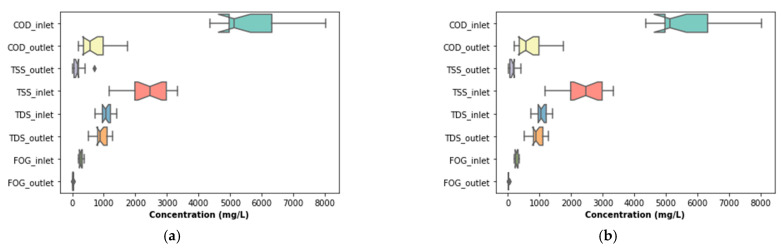
Boxplots of the DEGBR highlighted features before and after outliers’ replacement: (**a**) Boxplots before outliers’ replacement; (**b**) boxplots after outliers’ replacement.

**Figure 14:**
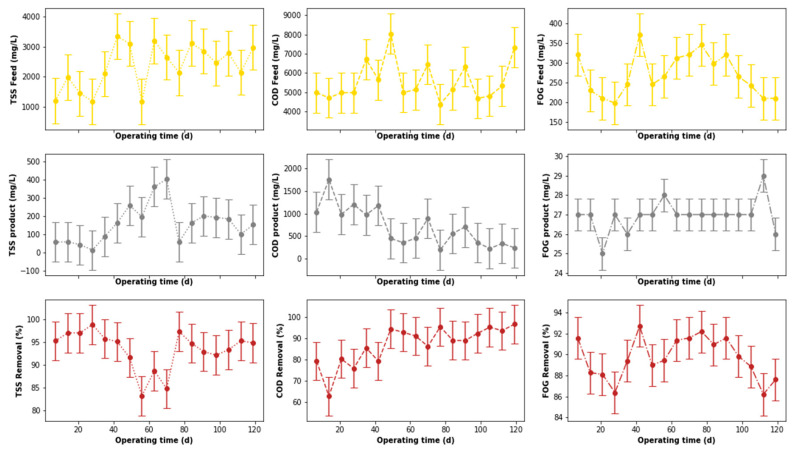
Performance of PSW DEGBR after outliers’ detection and replacement.

The output from the pre-treatment stage was separated into two streams. The first was supplied to the DEGBR, and the second to the EGSB. The output from both bioreactors was further treated separately using MBR systems receiving AD-treated effluent from both system types. Therefore, this section of the study evaluated the performance of the DEGBR as a secondary stage for the treatment of PSW. The variation of the assessment parameters used to conduct this evaluation is depicted in Figure 12, whereby the removal efficiencies above 65 to ~95% were noticed for each water quality parameter evaluated (COD, FOG, and TSS), which exceeded the FOG removal attained by previous studies [8]. To further consolidate these observations, the presence of outliers was also evaluated using boxplots, as depicted in Figure 13a, whereby the presence of outliers in the distribution of TSS concentrations was observed in the outlet stream, while for FOG concentrations, outliers were observed in the inlet stream. As in the previous section, these outliers were replaced by the median of the individual quality parameter distribution to produce distributions without outliers, as depicted in Figure 13b. The replacement of these outliers resulted in a reliable distribution depicted in Figure 14.

A comparison of the removal efficiencies varied throughout the study (see Figure 14) with indications that there was a slight change in the removal efficiencies between days 40 to 75 for the TSS removal. The alteration of the performance of the DEGBR for the removal of TSS was reduced during days 56 to 70. An improvement in the performance of the DEGBR after the outlier replacement was also noticed with the removal of FOG during days 14 to 28, and 102 to 109. At this stage it was not clear as to the reasons why the observed changes were observed, which is an indication that further analyses are required.

**Figure 15:**
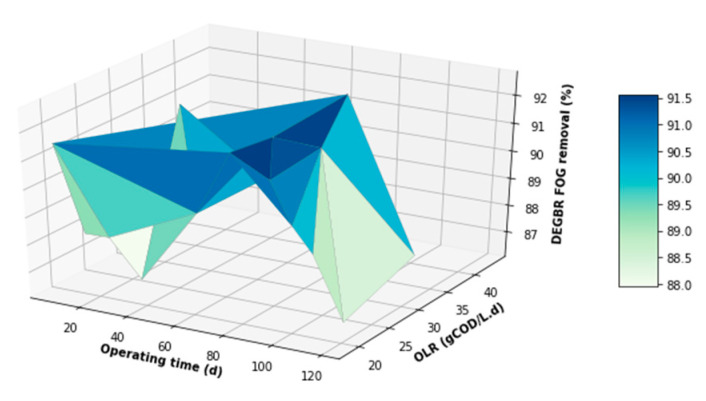
DEGBR FOG removal with respect to the operating time and the OLR.

**Figure 16:**
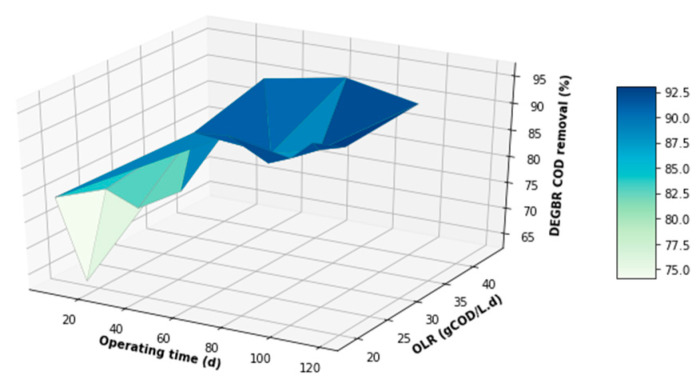
DEGBR COD removal with respect to the operating time and the OLR.

**Figure 17:**
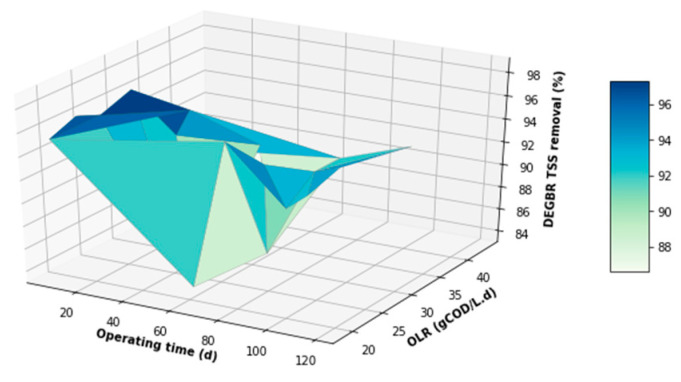
DEGBR TSS removal with respect to the operating time and the OLR.

The performance of the DEGBR was further assessed for relatedness, as shown in Figure 15, Figure 16 and Figure 17, which showed the response of the DEGBR in terms of the removal of the FOG, COD, and TSS, respectively, when a variation of the OLR was implemented in the experiment. A comparison of these graphs shows a better response of the DEGBR for the removal of COD, despite the various fluctuations of the OLR varying from ~18 to ~45 gCOD/L.d. Despite a good consistency in the evolution of the COD removal efficiencies, the DEGBR performance was higher at the beginning of the process for both the TSS and FOG removal efficiencies, as displayed in Figure 16 and Figure 17, with ranges varying between ~84 and ~98% for the TSS removal, and ~85% and ~93% for the FOG removal, which is an indication that the AD bed acted as a bio-filter, perhaps with some hydrolysis capacity.

**Figure 18:**
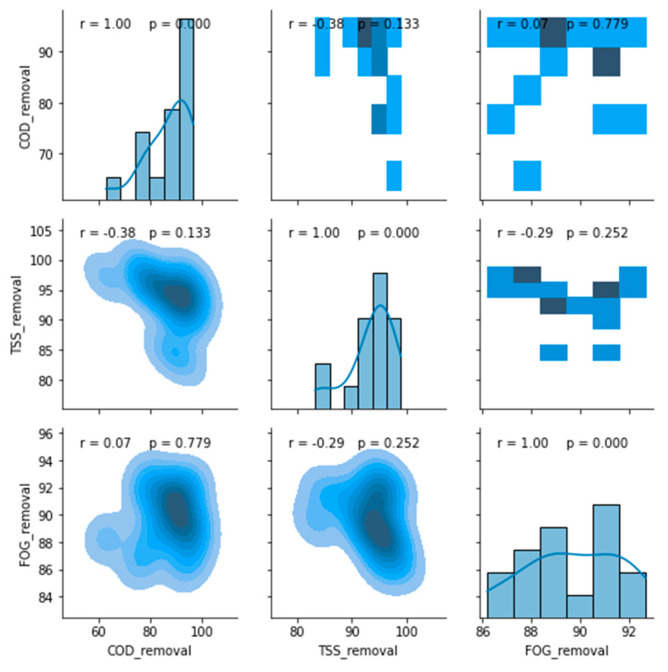
Correlation matrix between removal efficiencies of the DEGBR.

**Figure 19:**
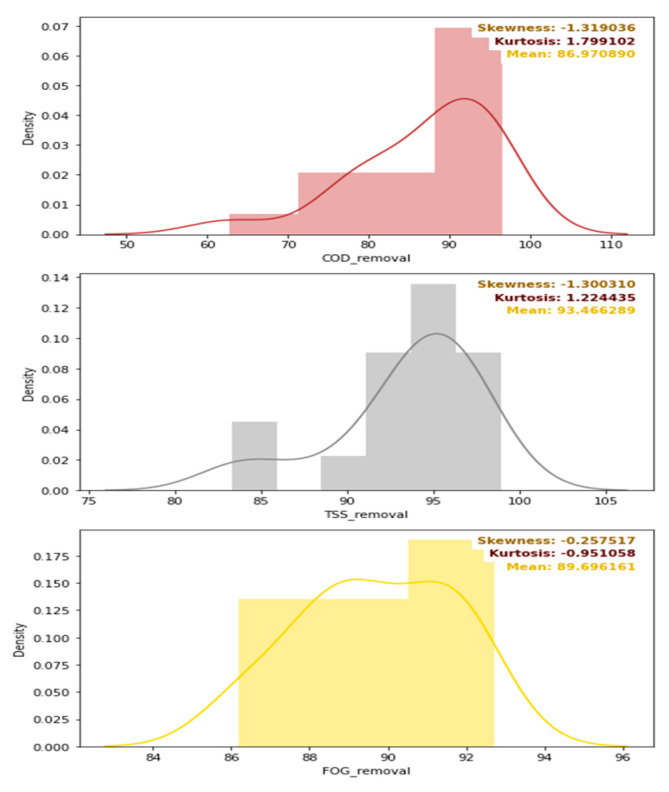
Density distribution, skewness, kurtosis, and mean values of the removal efficiencies of the DEGBR.

A feasible correlation between these removal efficiencies from the DEGBR treatment was evaluated using a correlation display, as illustrated in Figure 18. The latter shows a minimal correlation between the COD, FOG, and TSS removal efficiencies, as demonstrated by the low Pearson’s correlation coefficients. A further evaluation of the quality of the distribution of each of these removal efficiencies as well as the mean of each distribution is depicted in Figure 19. From the latter, it can be seen that the DEGBR performed the best for the removal of TSS with a mean removal percentage value of 93%, followed by the FOG mean removal percentage with an averaged value of ~90%, and the COD mean removal percentage with a value of ~87%. The distribution of the removal efficiency values was more skewed and tailed for the TSS and the COD removal, and less skewed and tailed for FOG removal; overall, the DEGBR displayed a good performance for the removal of these contaminants, which were further removed in the post-treatment stage using an MBR.

With reference to the reactor limitations and rationale mentioned in Table 2, the observed DEGBR performance displayed an improved performance with no signs of clogging nor need for backwashing [13,14,22].

#### 3.2.1. DEGBR-MBR Post-Treatment

**Figure 20:**
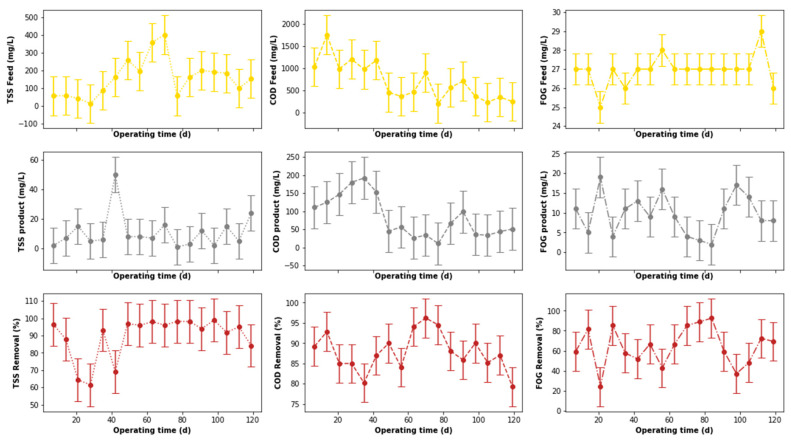
Performance of the MBR after the DEGBR treatment.

**Figure 21:**
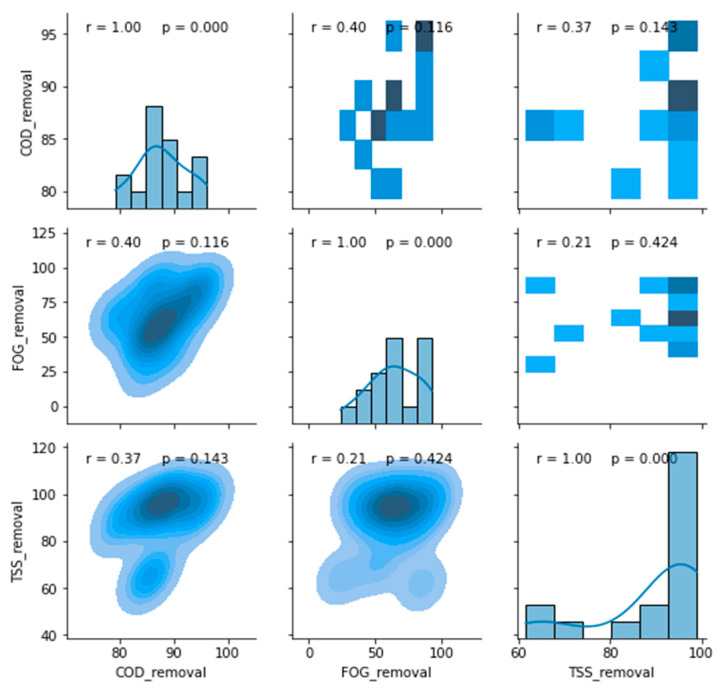
Correlation matrix between removal efficiencies of the MBR after DEGBR treatment.

A post-treatment stage consisting of an MBR was coupled to the DEGBR to further decrease the concentration of contaminants from the PSW. Figure 20 provides the variation of the concentration of the TSS, COD, and FOG both at the inlet and outlet of the MBR throughout the study. The boxplots of the assessed parameters reveal that there were no outliers in each distribution investigated, which indicated the consistency in the performance of the MBR unit. The removal efficiency values of the TSS remained consistently high after day 42 of operation, while an inconsistent variation was noticed for the COD and the FOG, albeit with minute variations. Similarly, to the DEGBR, a minimal correlation was found between these removal efficiencies, as depicted in Figure 21.

**Figure 22:**
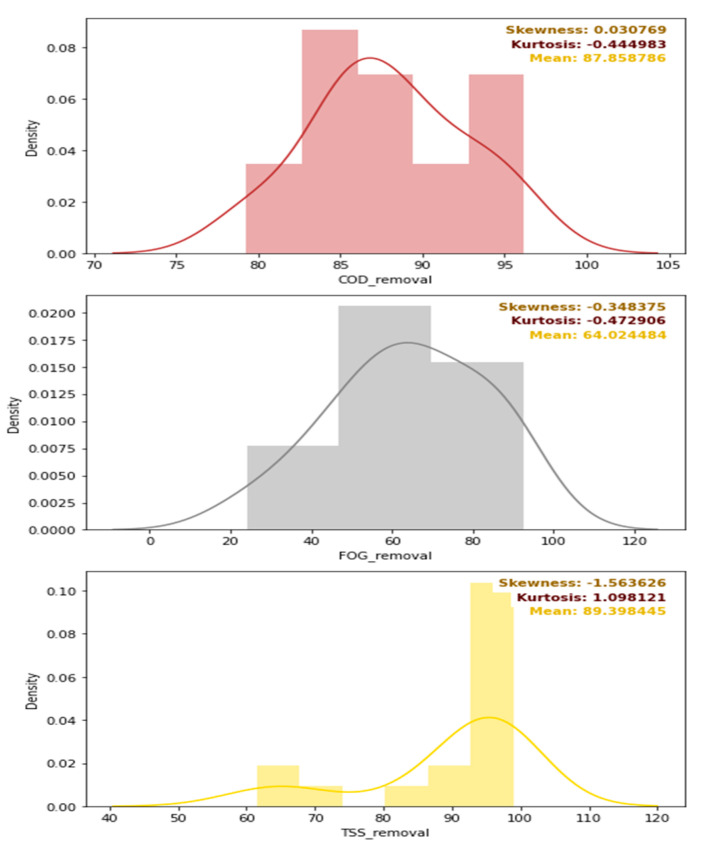
Density distribution, skewness, kurtosis, and mean values of the removal efficiencies of the MBR after DEGBR treatment.

**Figure 23:**
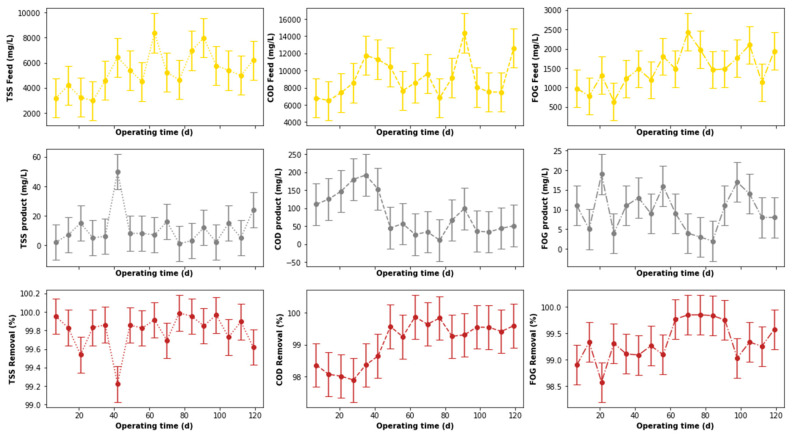
Overall performance of the combination of the pre-treatment stage, the DEGBR, and the MBR.

Figure 22 provides the density distribution, the skewness, the kurtosis, and the removal efficiency values from the MBR unit. A comparison between Figure 19 and Figure 22 shows that the DEGBR performed better than the MBR unit in terms of removal efficiency of the TSS and FOG. The MBR unit performed slightly better than the DEGBR for the mean removal efficiency of the COD, indicating its suitability as a polishing stage, i.e., the MBR unit performance significantly improved the quality of the treated PSW and contributed to the improved overall performance of the system composed of the pre-treatment stage, the DEGBR, and the MBR units. As shown in Figure 23, it can be observed that the performance of the overall process was >98% throughout the study.

#### 3.2.2. Overall Performance of Pre-Treatment-DEGBR-MBR System

**Figure 24:**
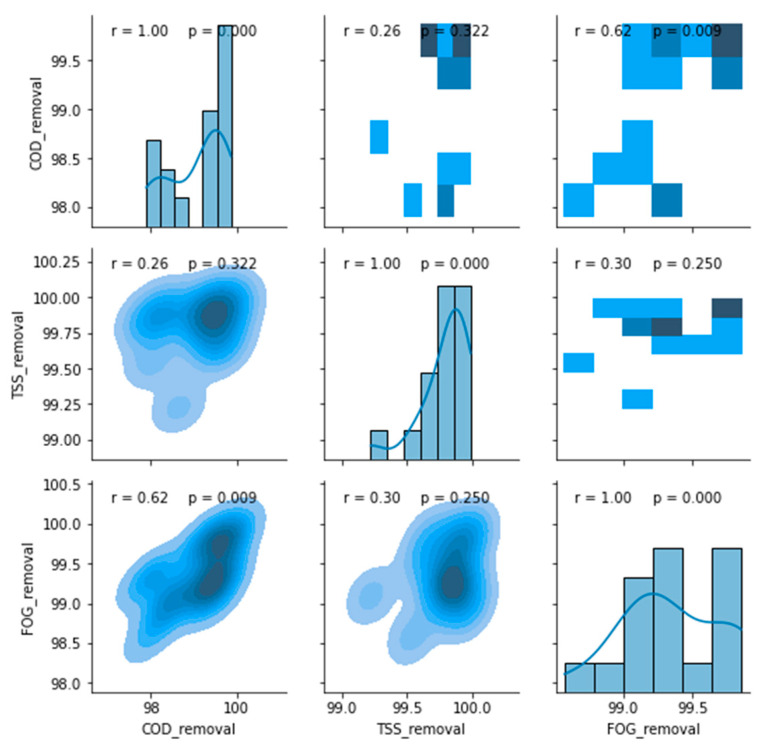
Correlation matrix between removal efficiencies of the combination of the pre-treatment stage, the DEGBR, and the MBR.

The overall performance of the integrated system combining a pre-treatment stage, the DEGBR, and the MBR is provided in Figure 23. This indicated a commendable performance of the system, with overall removal efficiency values of the FOG, COD, and TSS being >98% throughout the study. Although, Figure 24 indicated a minimal correlation between the removal efficiency of the FOG, COD, and TSS in the overall system, highlighting the suitability of such a system for the treatment of PSW or similar type of wastewater. The integration of different stages in the process addresses the shortcomings that one stage may have, and this can lead to overall potable water savings and perhaps the reuse of the treated water for other purposes such as irrigation—which, at this stage, still needs to be evaluated.

**Figure 25:**
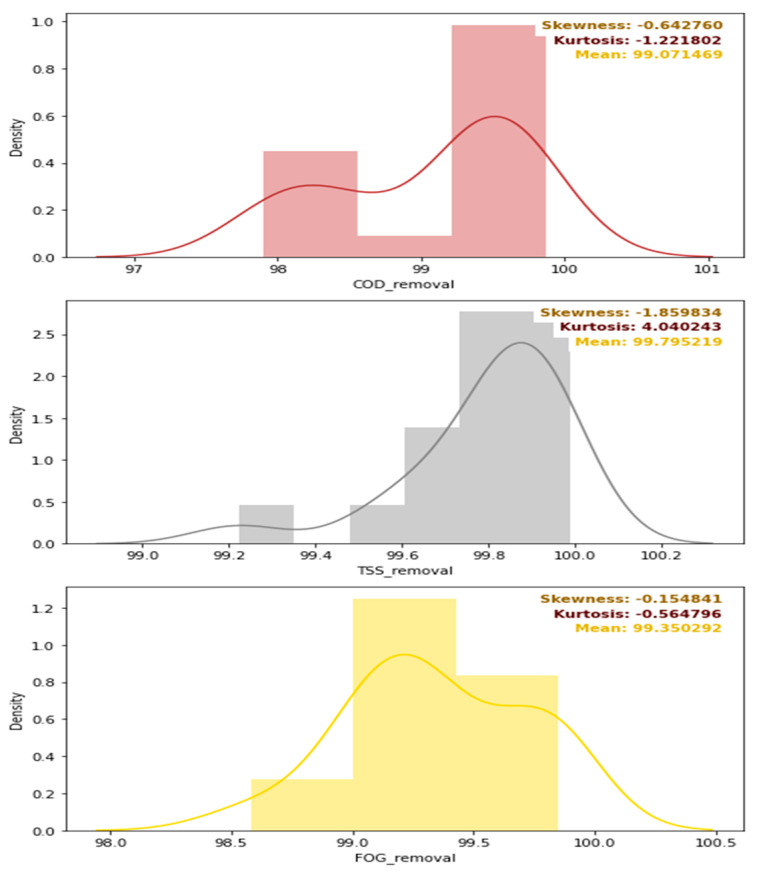
Density distribution, skewness, kurtosis, and mean values of the removal efficiencies of the combination of the pre-treatment stage, the DEGBR, and the MBR.

Figure 25 provides the density distribution, the skewness, the kurtosis, and the removal efficiency values of the combination of the pre-treatment stage, the DEGBR, and the MBR. A comparison between Figure 19 and Figure 22, Figure 25 shows that the increased removal efficiency of the combined pre-treatment-DEGBR-MBR approach which resulted in FOG, TSS and COD removal higher than 99%.

**Figure 26:**
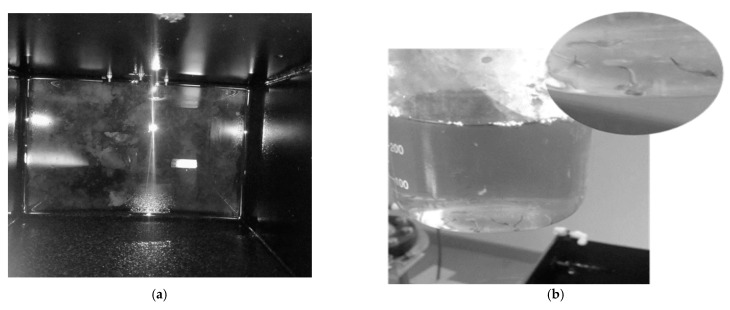
(**a**) Biofilm at the bottom of the membrane; (**b**) Membrane sample during treatment.

Despite these results signifying a positive membrane unit functionality, circumventing the typical membrane challenges such as clogging and membrane fouling, there was evident foam build-up during aeration. Furthermore, biofilm formed at the bottom of the membrane unit when an EGSB was used (shown in Figure 26a). The MBR operation was stopped after operating for 5 weeks due to signs of extracellular polymeric substances (EPS) build-up, as shown in Figure 26b. Thus, validating a re-evaluation of the sludge retention times (SRT) and aeration rate cited in some MBR operations [26] with excessive aeration being required, while extremely low SRTs were required due to the increased EPS in the MBR downstream. This presented a challenge that needs to be addressed in subsequent studies.

## 4. Conclusions

The use of a commercially viable bacterial suspension has provided a significant impactful FOG remediation strategy for PSW treatment, especially with regard to FOG removal. The study investigated the effectiveness of the implemented bio-physico-pre-treatment process, which has been proven to show a significant FOG concentration removal of 80%, which, in turn, aided the AD reactor performance.

Furthermore, this study also evaluated the Pre-treatment-DEGBR-MBR unit set-up in the form of a laboratory-plant for PSW treatment. The DEGBR achieved effective PSW containment removal at an HRT of 5.4 h and OLR range of ~18–~44 g COD/L·h. Moreover, the addition of the tertiary MBR stage offered a further treatment opportunity, which was achieved by a removal amount of 99% for COD, TSS, and FOG. The resultant effluent exceeded the set standard for effluent discharge. The system can be recommended as an effective solution for voluminous bird slaughtering industries.

## Figures and Tables

**Table 1:** Achieved effluent quality from past studies for PSW treatment.

Reactor Technology	Year of Development	Reactor Development Rationale and Challenges	Performance Results	Reference
Expanded Granular Sludge Bed (EGSB)	-	Side recycle stream improves efficiency due to the increase in sludge expansion. Up-flow configuration resulted in sludge washout during high FOG and TSS loading, thus resulting in methanogen loss, which reduced biological degradation.	65% tCOD removal	[1]
Static Granular Bed Reactor (SGBR)	2000	An SGBR with a down flow configuration was developed. Challenges include clogging of the underdrain. Hence, requiring periodic alleviation through backwash, disturbing the underdrain and granular bed build-up.	95% COD removal, 95% TSS removal, 90% FOG removal	[13,14]
Down-flow Expanded Granular Bed Reactor (DEGBR)	2019	A hybrid of EGSB and SGBR, with down/top feeding configuration and a recycle.	99,6% COD removal, 93,7% FOG removal	[8]

**Table 2:** Developed pre-treatment technologies and their COD, FOG, and TSS removal efficiency.

Pre-Treatment Technology	Reactor Coupled with the Pre-Treatment Unit	Limitations	Performance Results	Study of Reference
Thermal Autoclaving	-	Additional physical separation of the clear liquid and semi solid sludge is necessary.	81% COD removal, 59% FOG removal, 43% TSS removal.	[14]
Chemical Dissolved Air Flotation (DAF)	UASB	System showed instability due to the varying PSW influent. Additionally, system efficiency is highly affected by chemical used.	43% ± 15% suspended solids (SS) removal and 49% ± 8% oil and grease (O&G) removal.	[15]
Biological Enzymatic Pre-treatment	-	Optimal enzyme dose is not yet established. Though the increase in dose results in increased free fatty acid and VSS treatment, the dose has an optimal point where higher doses do not contribute to increased effectiveness.	Increase in free fatty acids and 10% hydrolysis promotion due to lipase, 88% COD reduction in PSW.	[13,16,17]
Hydrodynamic Cavitation	-	Optimum conditions included addition of Fenton reagent.	Increased COD treatment to 44.2%. Biological oxidation treatment time reduction from 60 to 36 h.	[18]

**Table 3:** PSW Characteristics.

Parameter	Units	Minimum	Maximum	Average	Reference	This Study
COD	mg/L	4100	9100	4317	[23]	6500–21,000
TSS	mg/L	1580	3750	2800 ± 950	[23,24]	2985–8363
FOG	mg/L	280	8228	1655 ± 1880	[8]	640–4500

**Table 4:** Sample Analysis Methods.

Parameter	Method
Temperature	EPA method 9040C
Total suspended solids (TSS)	EPA method 160.2
Total chemical oxygen demand (tCOD)	EPA method 410.4
Fats, oils, and grease (FOG)	EPA method 10056

## Data Availability

Not applicable.
